# Cross-Species Insights into Autosomal Dominant Polycystic Kidney Disease: Provide an Alternative View on Research Advancement

**DOI:** 10.3390/ijms25115646

**Published:** 2024-05-22

**Authors:** Jianing Luo, Yuan Zhang, Sakthidasan Jayaprakash, Lenan Zhuang, Jin He

**Affiliations:** 1 College of Animal Sciences, Zhejiang University, Hangzhou 310027, China; luojianing@zju.edu.cn (J.L.); zhangyuan2020@zju.edu.cn (Y.Z.); zhuangln@zju.edu.cn (L.Z.); 2 Department of Biotechnology, Hindustan Institute of Technology and Science, Tamil Nadu 603103, India; jsakthi88@gmail.com

**Keywords:** ADPKD, cross-species research, therapeutic strategies

## Abstract

Autosomal Dominant Polycystic Kidney Disease (ADPKD) is a prevalent hereditary disorder that affects the kidneys, characterized by the development of an excessive number of fluid-filled cysts of varying sizes in both kidneys. Along with the progression of ADPKD, these enlarged cysts displace normal kidney tissue, often accompanied by interstitial fibrosis and inflammation, and significantly impair renal function, leading to end-stage renal disease. Currently, the precise mechanisms underlying ADPKD remain elusive, and a definitive cure has yet to be discovered. This review delineates the epidemiology, pathological features, and clinical diagnostics of ADPKD or ADPKD-like disease across human populations, as well as companion animals and other domesticated species. A light has been shed on pivotal genes and biological pathways essential for preventing and managing ADPKD, which underscores the importance of cross-species research in addressing this complex condition. Treatment options are currently limited to Tolvaptan, dialysis, or surgical excision of large cysts. However, comparative studies of ADPKD across different species hold promise for unveiling novel insights and therapeutic strategies to combat this disease.

## 1. Introduction

**Figure 1:**
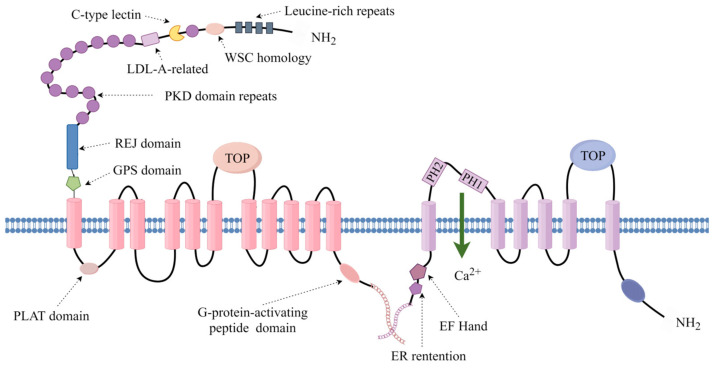
Schematics of PC-1 and PC-2 with labeled domains. The schemas of PC-1 and PC-2 illustrate their complex structures and key domains. Both proteins interact through coiled-coil domains in their tails, critical for calcium signaling, which is disrupted in polycystic kidney disease. The pictures in this paper were created through the FigDraw website, https://www.figdraw.com/static/index.html#/ (accessed on 11 January 2024).

In humans, numerous hereditary diseases, including Autosomal Dominant Polycystic Kidney Disease (ADPKD), can induce the development of renal cysts. In ADPKD, the primary pathogenic genes are typically *PKD1* and *PKD2*, responsible for encoding Polycystin-1 (PC-1) and Polycystin-2 (PC-2), respectively. PC-1, with an approximate size of 460 kDa [1], presents as a membrane protein characterized by an extended N-terminal extracellular domain, 11 transmembrane domains, and a short intracellular C-terminal domain (Figure 1). The predominant localization of PC-1 spans in primary cilia of cells, tight junctions of cell membranes, desmosomes, and adhesion plaques [2]. In contrast, PC-2, smaller in size at around 110 kDa, harbors six transmembrane domains with intracellular N and C terminals (Figure 1). As a member of the transient receptor potential channel superfamily, PC-2 is situated in primary cilia, centrosomes, and the endoplasmic reticulum [2]. Functionally, PC-2 serves as a Ca^2+^ permeable non-selective cation channel.

**Figure 2:**
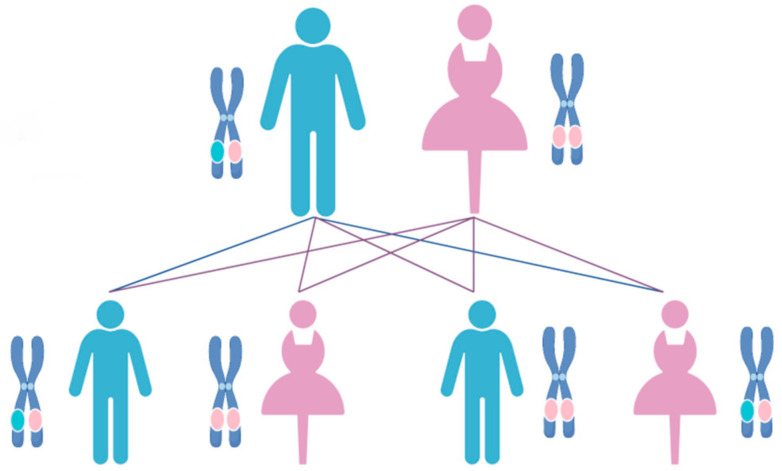
Hereditary mode of ADPKD. Autosomal dominant inheritance is a genetic pattern in which a trait or disorder is passed down through families. In this pattern, one copy of the altered gene in each cell is sufficient to increase the risk of developing a condition. Autosomal dominant conditions can occur in every generation of an affected family, and the risk of passing the condition from an affected parent to a child is 50%. Blue dots: normal genes; pink dots: pathogenic genes. The pictures in this paper were created through the FigDraw website, https://www.figdraw.com/static/index.html#/ (accessed on 13 January 2024).

ADPKD presents as a systemic disorder marked by the growth of numerous cysts within both kidneys. In addition to renal manifestations, ADPKD encompasses a range of extrarenal pathologies and symptoms, including the emergence of liver cysts, seminal vesicle cysts, pancreatic cysts, and hypertension, among other complications [3,4,5]. Cyst formation within the kidneys predominantly originates from the renal tubules and collecting ducts. The fluid composition within these cysts mirrors plasma, containing vital constituents such as Na^+^, K^+^, Cl^−^, H^+^, creatinine, and urea. As the disease advances, cyst enlargement exerts continuous pressure on adjacent renal tissues, triggering cytokine activation and the recruitment of inflammatory cells, ultimately leading to interstitial inflammation and fibrosis [6]. Progressive decline in renal function culminates in renal insufficiency and eventual progression to end-stage renal disease, necessitating interventions such as dialysis or kidney transplantation. Furthermore, ADPKD represents a chronic condition with variable progression among individuals and species, posing diagnostic and management challenges. This variability underscores the considerable risk of misdiagnosis among susceptible patients. Symptoms resembling ADPKD have been documented in various animal species, including dogs, cats, pigs, cattle, and sheep [7,8,9,10,11,12,13,14,15]. Affected animals exhibit notable similarities in inheritance patterns, age of onset, clinical symptoms, and pathological features (Figure 2). The presence of genetically akin diseases across diverse species may be attributed to mutations in homologous genes [16].

The precise mechanisms governing the initiation and development of ADPKD remain incompletely elucidated. However, numerous models have been proposed to elucidate the pathogenesis of ADPKD and the mechanisms by which mutations in the *PKD1* and *PKD2* genes lead to the development of polycystic kidneys. These include the “two-hit” (carry a germline mutation with somatic mutations) and “third hit” (external factor stimulation) theories, the over-expression model (the expression of polycystic protein increased), the haploinsufficiency model (the expression level of the gene is reduced to 50% of the usual level), as well as the trans-heterozygosity (the expression of one allele related to cyst formation is reduced to 50% and a mutation occurs in another related gene within the somatic cell) and threshold models (expression above or below the steady-state threshold), which have been substantiated through experimental studies in mice [17,18,19,20,21,22]. Nevertheless, several signaling pathways are implicated in its pathogenesis, consisting of the HIF-1α, mTOR, TSC-1/2 complex, Wnt, JAK/STAT, AMPK, and PPAR-γ pathways [23,24,25,26,27,28,29]. In addition, the dysregulation of Ca^2+^ channels and cAMP signaling has been observed as contributing to the accelerated proliferation of cystic epithelial cells [30]. In vitro data and animal models of non-human origin have demonstrated a reduction in autophagy flux in ADPKD [31]. Furthermore, current studies indicate that metabolic disorientation emerges as a pivotal characteristic of ADPKD, with affected individuals predisposed to aberrant metabolic reactions or accumulation of metabolites, exacerbating disease progression [32].

## 2. Comprehensive Investigation of Multi-Species Epidemiology

ADPKD represents a widespread condition globally, standing as the most prevalent form of polycystic kidney diseases. Being autosomal dominant inheritance in nature, ADPKD exerts its impact on millions of people worldwide. Present estimation indicates that around 12 million individuals globally are inflicted with ADPKD [33], with approximately 1.5 million cases documented in China alone [34]. As lifestyles shift with aging and altered habits, the incidence of this malady is expected to surge further, while prevalence rates manifest variability across distinct regions and among diverse ethnic cohorts [35]. Thus, ADPKD imposes a pressing global health concern, affecting approximately 1 in 400 to 1 in 1000 individuals. This condition manifests across both adult and pediatric populations without evident gender bias. The presence of a familial history of ADPKD significantly escalates the risk of other family members to develop the disease. In Europe, ADPKD ranks as the fourth most prevalent cause of kidney disease necessitating dialysis or replacement therapy, with one in ten patients requiring renal replacement therapy due to ADPKD [35,36]. In China, data gleaned from Shanghai’s registration records reveal that ADPKD accounts for 4.7% of end-stage renal disease (ESRD) cases [34], emerging as a principal cause of end-stage renal failure, accounting for 10% of all cases [33].

In addition to humans, ADPKD or other related polycystic kidney diseases emerge as a frequently encountered hereditary kidney disorder in cats to date [37,38,39]. Currently, the *PKD1* gene has been pinpointed as the primary cause of the ADPKD in cats, yet the presence of additional mutations potentially influencing feline polycystic kidney diseases remains an area of investigation [40,41,42]. Polycystic kidney disease is prevalent among adult Persian cats and breeds with Persian lineage, encompassing cherished breeds like the Garfield and other hybrid varieties. However, ADPKD is not confined solely to Persian-related felines; occurrences have also been reported in British shorthairs, American shorthairs, exotic longhairs, kinjiras, maines, German curlys, and various other cat breeds [43,44]. Globally, an estimated 40% to 50% of Persian cats, comprising approximately 6% of the worldwide feline population, are afflicted with ADPKD [45,46,47,48], affecting both sexes without marked gender preference [49].

While a number of reports have addressed polycystic kidney disease in dogs [50,51,52,53,54], a conclusive genetic link to ADPKD remains elusive. Inbreeding practices within specific dog breeds have increased the likelihood of harboring the mutated gene. Notably, breeds such as the West highland white terrier, bull terrier, cairn terrier, and various other terrier breeds exhibit heightened susceptibility, although the instances of polycystic kidneys have also been noted in other breeds, including shepherd dogs. In Australia, bull terrier polycystic kidney disease (BTPKD) demonstrates particularly high incidence rates, particularly among dogs showcased in pet exhibitions and breeding establishments. Data summarization indicates a comparable incidence between male and female dogs, with no discernible gender bias observed [55].

Polycystic kidney disease was also seen in the domesticated animals such as pigs, cattle, and sheep. Clinically diagnosed cases in these animals are rare, and it is typically identified post-mortem during slaughter, revealing kidneys riddled with numerous cysts. Studies estimate that approximately 26% of cattle and 47% of pigs exhibit renal cysts [56,57]. The restricted breeding practices in livestock, characterized by a small number of selected paternal and maternal lines, result in nearly half of the offspring inheriting kidney cysts. However, since most livestock are slaughtered at a young age, the potential risks associated with this disease are often overlooked. However, considering the impact of ADPKD on human fertility [58], it is reasonable to speculate that, in addition to kidney cyst formation, cysts may also arise in the seminal vesicles of livestock. This could potentially compromise the reproductive performance of breeding animals, leading to diminished sperm production and quality, as well as reduced fertilization and conception rates. Consequently, this may lead into lower pregnancy rates among females, an increased risk of miscarriage, fewer newborn offspring, and the birth of weaker, malformed young animals. Such outcomes markedly escalate the expenses associated with breeding while diminishing production efficiency. Therefore, it is imperative to conduct research into ADPKD in livestock to gain a deeper understanding of the disease and address these potential challenges.

Regardless of the species—such as human, dog, cat, pig, or other animals—the actual prevalence of ADPKD is often considerably higher than commonly perceived due to the absence of early diagnosis and effective reproductive management. If left unaddressed, this could result in a more widespread occurrence of ADPKD. Therefore, it is imperative to screen and test individuals with a family history of the disease and animals exhibiting related symptoms to detect ADPKD at the earliest opportunity. Moreover, genetic testing should be prioritized in animal breeding programs to mitigate the propagation of the polycystic kidney gene, thereby reducing the incidence of ADPKD.

## 3. Pathological Features and Cross-Species Manifestations of ADPKD

ADPKD is an inherited condition that affects various species. Despite variations in symptoms and severities across different species, there are common pathological characteristics. The hallmark of ADPKD is the formation of cysts of varying sizes in both kidneys, which can be found across the renal cortex and medulla. Additionally, a subset of patients may develop cysts in the liver, potentially progressing to severe Polycystic Liver Disease (PLD). The condition exhibits a higher prevalence in females, while male patients typically exhibit cysts in the kidneys and seminal vesicles. Moreover, cysts can also occur in the pancreas, spleen, thyroid, and other areas (Table 1) [57,58,59,60]. Experimental research has shed light on the role of *Pkd1* gene variations in the development and severity of ADPKD in mice [61]. Mice possessing a typical *Pkd1* gene remain disease-free, while the presence of even a single, less potent variant of the *Pkd1* gene can trigger swift cystic changes within the kidneys. Furthermore, mice with two copies of such gene variants exhibit a gradual onset of renal cysts. These observations underscore the critical influence of PC1 protein dosage, which is encoded by the *Pkd1* gene, on disease severity. The phenotype of ADPKD intensifies as the levels of PC1 protein decrease, thereby highlighting the profound effects of gene dosage on the disease’s manifestation. Collectively, these insights substantiate the pivotal role of gene dosage effects in the pathogenesis of ADPKD. As the disease progresses, these cysts gradually enlarge, exerting pressure on surrounding normal tissues and causing local tissue damage. Histological examination of the kidneys in ADPKD patients reveals significant interstitial inflammation, characterized by a high infiltration of inflammatory cells into the interstitium (Table 1). Immunohistochemical analysis of kidneys from advanced ADPKD patients demonstrates increased expression of fibrosis markers such as α-SMA, indicating interstitial fibrosis. Moreover, there is a decrease in the expression of E-cadherin, a marker of epithelial cells, alongside an increase in N-cadherin, a mesenchymal marker, suggesting a dedifferentiation of renal epithelial cells and consequent changes in cell function [62]. Furthermore, the disease is characterized by epithelial-mesenchymal transition (EMT) and excessive accumulation of extracellular matrix (ECM) (Table 1) [62].

In the advanced stages, renal tissue undergoes atrophy, healthy renal parenchyma is gradually replaced, and ultimately leads to chronic renal failure. At this stage, both kidneys may increase in volume, often assuming irregular shapes. While symptoms may be subtle in early stages, as the disease progresses to intermediate or late stages, kidneys gradually lose normal physiological function due to the relentless compression of healthy tissue by expanding cysts. Severe cases result in irreversible renal failure, accompanied by complications such as azotemia, hyperphosphatemia, acidosis, and anemia. Additionally, studies indicate that ADPKD patients and their families face an increased risk of heart disease, including aortic valve malformation and aortic root dilation [63], this is due to the fact that polycystin proteins also play an important role in the development and maintenance of the cardiovascular system, and their abnormalities may indirectly affect the cardiovascular system, leading to vascular dysregulation, which significantly impacting their quality of life and posing life-threatening risks. In addition, most patients in the early stages of ADPKD exhibit normal renal function; however, hypertension prevalence can escalate to as high as 50%. Hypertension will also heighten the risk of cardiovascular disease. Without the timely control of ADPKD, the condition will persist in deteriorating, resulting in a continuous rise in blood pressure. This imposes a significant burden on both the heart and kidneys, hastening the progression towards renal failure. ADPKD-like disease in dogs and cats closely resembles that in humans (Table 1 and Table 2). The majority of cysts were formed within the renal cortex and medulla, with the quantity and size varying based on individuals and distinct breeds [64]. Additionally, cysts may form in the liver, pancreas, uterus, and abdominal cavity. Some dogs and cats may exhibit focal proliferation of renal tubular epithelium and develop heart disease, particularly the Bulldogs, who appear to have an elevated risk for cardiac issues [65]. Moreover, in cats, chronic renal failure has been linked to hypertension [66,67,68,69]. Studies comparing blood pressure indices in normal cats versus those with mild and severe ADPKD symptoms have shown that cats with ADPKD have an increased average arterial pressure compared to the control group [70]. Histological examination, ultrastructural analysis, and lectin staining have indicated that the cysts most likely originate from the nephron segments and collecting ducts. BrdU-based proliferation studies have revealed a higher proliferation rate of cyst lining epithelial cells in some ADPKD-affected kittens compared to healthy kittens [64]. Additionally, research has delved into the histological and ultrastructural characteristics of renal lesions in BTPKD, identifying that cysts in BTPKD may arise from either nephrons or collecting ducts [71,72]. Initially, most young animals exhibit minimal symptoms, typically presenting with small cysts in limited numbers, while renal function remains relatively unaffected. However, in adult or elderly dogs and cats, cysts significantly increase in size and quantity, often accompanied by renal dysfunction. As time progresses, the condition of affected dogs and cats deteriorates gradually, culminating in end-stage renal disease. Nevertheless, some dogs and cats may remain asymptomatic for most of their lives, only experiencing renal failure in later stages (usually after the age of 7). Cats, in particular, tend to develop renal failure earlier than dogs and may succumb to other ailments before renal failure becomes a significant factor.

In livestock, such as pigs, cattle, and sheep, cyst formation typically occurs in the kidneys and liver, leading to the enlargement of these organs (Table 1). Some animals may also develop ascites, wherein affected piglets accumulate significant amounts of fluid in their abdomens, resulting in pronounced distension. Both kidneys of the livestock are usually affected, exhibiting a marked increase in volume compared to healthy organs. Upon sectioning, numerous cysts are visible, permeating throughout the medulla and cortex, creating a honeycomb-like structure [73]. Conversely, the affected liver presents a greenish-brown hue, displaying evident bile duct hyperplasia and containing numerous irregularly shaped cystic vesicles filled with fluid. Microscopic examination of the kidneys using Hematoxylin and Eosin (H&E) and Masson’s trichrome staining methods reveals that the cysts exert pressure on the surrounding cortex and medulla (Table 2). These cysts are lined by a layer of flattened epithelial cells, bordered by fibrous connective tissue that often extends and replaces the renal parenchyma. In these regions, renal tubules undergo atrophy and degeneration. While ADPKD in livestock may not attract the same societal attention as sudden events like infectious epidemics, its impact on the health and production efficiency of livestock is significant and cannot be overlooked. In humans, ADPKD exhibits considerable clinical diversity not only among different families but also among members of the same family. This diversity is evident in the disease’s manifestations, its severity, and the age at which it becomes apparent, resulting in a spectrum of disease experiences even among individuals sharing identical genetic predispositions. Although ADPKD is the most prevalent hereditary kidney condition in adults, the age of disease onset varies, with some individuals experiencing early onset forms, while others have a later onset.

The alignment of symptoms across diverse species hints at the probability of shared gene mutations underlying ADPKD. Unraveling the differences and connections about ADPKD across various species can enrich our comprehension of the molecular mechanisms and genetic traits associated with this condition. Through syntheses and analyses of these data, we can pinpoint commonalities that aid in identifying pivotal genes and pathways involved in the disease’s pathogenesis. This interdisciplinary research methodology promises invaluable insights and direction for the prevention and treatment of ADPKD, furnishing us with a more robust toolkit to address the challenges posed by this condition.

## 4. Molecular and Genetic Mechanisms of ADPKD across Species

In humans, ADPKD is usually caused by mutations in two genes, *PKD1* and *PKD2*, and the presence of a mutated gene in an individual can precipitate symptom manifestation [74]. Most of the ADPKD cases arise from inherited mutated genes from parents, with only around 10% stemming from de novo mutations. *PKD1* and *PKD2* encode PC-1 and PC-2, respectively and it is recognized that several proteins can interact with PC-1 or PC-2, thereby regulating diverse signaling pathways [75]. Both PC-1 and PC-2 harbor N-linked glycosylation modification sites, and ALG8, ALG9 also undergo glycosylation within the endoplasmic reticulum. GANAB (neutral α-glucosidase AB) is an α-glucosidase primarily responsible for the catalysis of hydrolysis reactions involving incomplete glucose moieties on the endoplasmic reticulum. Furthermore, the genes encoding these proteins have been reported as one of the potential pathogenic loci for ADPKD. Studies have suggested that GANAB and PC-1 can confer PC-1 stability through interaction.

**Figure 3:**
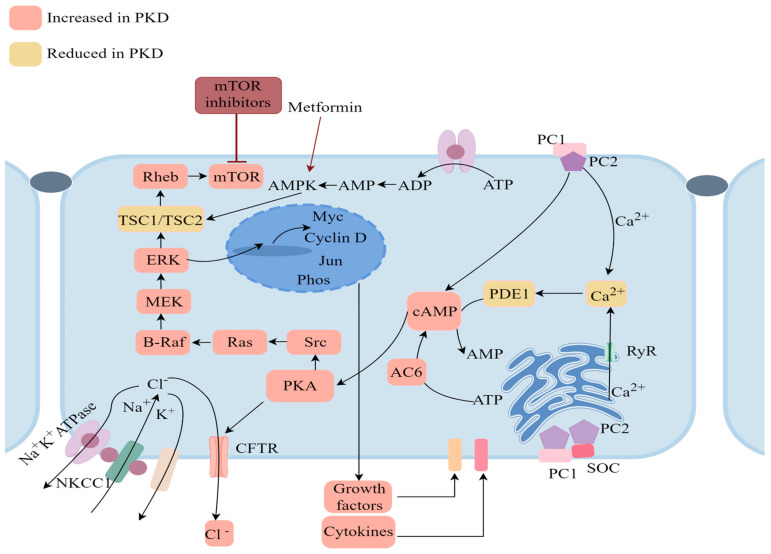
Molecular mechanism of ADPKD. In ADPKD, altered levels of Ca^2+^ and elevated cAMP contribute to disease progression. Dysregulated calcium signaling can lead to increased cell proliferation and changes in extracellular matrix metabolism, promoting kidney cyst growth. Concurrently, increased cAMP levels activate PKA, which further stimulates cell proliferation and extracellular matrix secretion. The interplay between dysregulated calcium and elevated cAMP creates a harmful loop that intensifies renal damage in ADPKD, offering potential targets for therapeutic intervention to normalize these pathways and stop disease advancement. The pictures in this paper were created through the FigDraw website, https://www.figdraw.com/static/index.html#/ (accessed on 21 May 2024).

PC-1 and PC-2 form unique heterotetrametric complexes (1 PC-1 + 3 PC-2) that can interact at the primary cilia of renal epithelial cells [75,76] through their C-terminal interactions, participating in the regulation of cell differentiation, proliferation, and apoptosis [77]. Studies have demonstrated that primary cilia function as sensory antennae, adept at detecting chemical and mechanical stimuli, including blood flow sensation in renal tubules, and are integral in maintaining renal epithelial cell homeostasis. Disruption of cilia has been implicated in renal cysts initiation [78]. These complexes play a role in regulating cellular calcium ion channel function, in which process ALG8 and ALG9 play important roles. Upon stimulation by abnormal fluid flow within the renal tubules, this channel can disrupt calcium-related pathways, leading to an imbalance in Ca^2+^ signaling (Figure 3). This disruption alters intracellular Ca^2+^ levels, impacting cAMP synthesis and degradation [79]. Fluctuations in cAMP levels can stimulate cell proliferation derived from cysts [80]. Furthermore, signaling pathways involving MEK, ERK, CREB, STAT, STAT6, and mTOR are upregulated (Figure 3), contributing to the repair and proliferation of damaged renal tissue [81,82]. Remarkably, mTOR plays a pivotal role in regulating cell growth and division, as well as inhibiting catabolic processes. mTOR promotes cell growth by inhibiting protein catabolism, such as through autophagy regulation [83]. A study has identified a decline in lysosomal acidification, LAMP degradation, and CTSB/tissue protease B processing and activity in *PKD1*-deficient mIMCD3 cells. This reduction disrupts autophagosome-lysosome fusion, decreases ubiquitination from multivesicular bodies (MVB)/exosomes to lysosomes, and increases the secretion of unprocessed CTSB into the extracellular space [84]. Moreover, mTOR can activate the PI3K/Akt pathway. When Akt is activated, it phosphorylates the GSK3 subtype at its highly conserved N-terminal regulatory site, thereby inactivating the kinase and regulating cell apoptosis and glucose metabolism through GSK3. Akt also influences cell apoptosis, growth, proliferation, metabolism, protein synthesis, and transcription by phosphorylating and inhibiting FoxO1/3a transcription and TSC2, and can reduce intracellular calcium levels [85]. Under conditions of low ATP levels, hypoxia, or DNA damage, the stress response metabolic regulatory factor AMPK is activated to inhibit TSC2 [83].

The Wnt signaling pathway plays a crucial role in cyst formation, influencing cell proliferation, differentiation, and apoptosis [86]. Dysregulation of this pathway can significantly contribute to cyst growth. Abnormal activation of Wnt signaling leads to increased proliferation and fluid secretion by renal epithelial cells, facilitating the continuous expansion and dissemination of cysts. Equally, beyond signaling irregularities within cystic structures, abnormal expression of TGF-β in renal fibrosis areas suggests a potential contribution of renal fibrosis to eventual renal failure in ADPKD [87]. In addition, abnormal oxidative stress and inflammatory responses can damage cells and tissues. In ADPKD, oxidative stress may accelerate the development of renal cysts by affecting cellular nucleic acids and proteins [88].

A vast amount of data is available on the mechanisms underlying human ADPKD, particularly through the research conducted in mice, which has yielded valuable insights into the underlying mechanism. However, there is a lack of study specifically examining the molecular mechanisms of ADPKD in dogs, cats, pigs, and other animal species. Human *PKD1* contains several polypyrimidine tracts, and many ADPKD patients exhibit deletion mutations in one or more polypyrimidine regions near the intron 21 and intron 22 [89]. In contrast, in other species, the polypyrimidine region may be absent or possess a different length and sequence composition. For example, mice and rats lack this region, and spontaneous ADPKD has not been reported in these rodent models. In the pigs, the polypyrimidine region may be involved in the regulation of gene expression, affecting the development, growth and diseases, although there is no exact research or evidence that the polypyrimidine region is directly related to its spontaneous PKD [90]. To date, no reported homozygous animals with *PKD1* or *PKD2* mutations exist, suggesting that such mutations may result in embryonic lethality. Thus, several heterozygous mutations in these animals have been detected. Specific mutation in the *PKD1* gene of cats has been identified (Table 3), where the adenine at the 3284th position in exon 32 is replaced by cytosine (C.10063C>A), accounting for approximately 30% of diseased Persian cats. This mutation introduces premature termination codons into the mRNA, leading to the loss of 25% of the C-terminus during the formation of PC-1. A linkage analysis study on seven Persian cat families revealed a significant association between the ADPKD phenotype and the FCA476 marker, which is located within the 10 cR region of the cat *PKD1* gene on the E3 chromosome [91]. This suggests that the *PKD1* gene or other genes within this region may be implicated in ADPKD in cats. In dogs, ADPKD is prevalent in certain Bulldog lines, resembling the condition observed in humans. The gene, situated on the dog chromosome 6, is linked to ADPKD and shares homology with the region of the human chromosome containing the *PKD1* gene. Studies have identified a mutation of *PKD1* gene G>A on dog chromosome 29 associated with BTPKD (Table 3). This mutation serves as a diagnostic tool to assess the risk of ADPKD in young and breeding dogs [92]. Additionally, the symptoms and pathological characteristics of ADPKD in pigs, cattle, and sheep closely resemble those in humans. However, due to restricted breeding within these domestic animal populations, offspring are more susceptible to inheriting pathogenic genes, increasing the genetic risk of ADPKD within specific populations. Regrettably, genetic localization studies targeting the polycystic kidney phenotype in domestic species like pigs, cattle, and sheep are currently lacking, representing a significant void in the existing research landscape.

The genetic models and molecular mechanisms of ADPKD across various species exhibit significant similarities, though with subtle variations likely stemming from shared gene mutations among different species. Consequently, cross-species comparisons can enrich our understanding of ADPKD pathogenesis in human and animals, providing clearer guidance for diagnosis and treatment strategies.

## 5. Diagnostic Approaches and Clinical Manifestations

While the exact pathogenesis of ADPKD remains elusive, continuous research and advancements in modern medical technology have facilitated its diagnosis through various methods. Clinical symptoms observed in dogs, cats, and other animals closely mirror those in humans, implying analogous diagnostic approaches.

**Figure 4:**
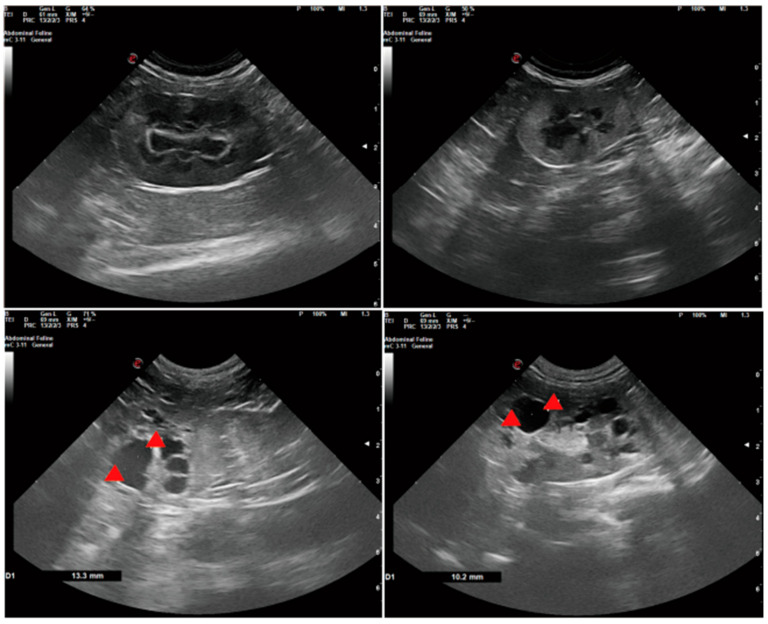
Ultrasonographic image of the felines’ kidneys. The first two images show the normal kidneys of felines. Red arrows indicate cysts in the kidney.

**Figure 5:**
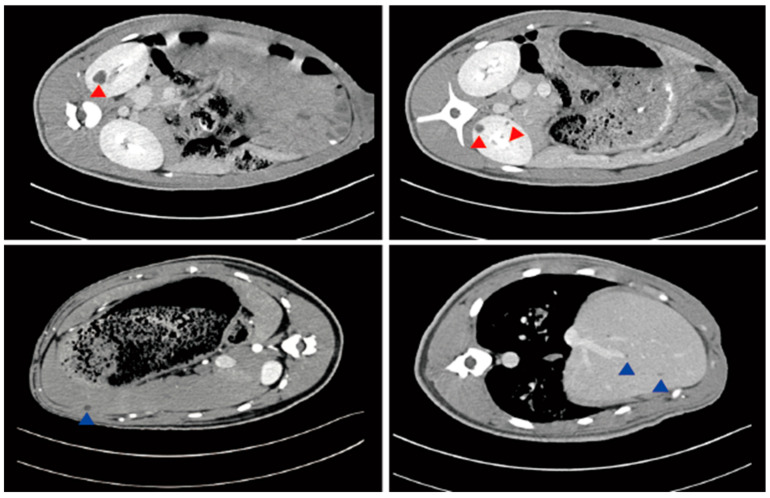
Contrast enhanced computed tomography of the pigs’ kidneys and livers. Red arrows indicate cysts in the kidney; blue arrows indicate cysts in the liver.

In clinical settings, early stages of ADPKD often manifest without significant changes in kidney size, with patients experiencing subtle symptoms like headaches, insomnia, and blurred vision (Table 4, Figure 4 and Figure 5). Hypertension emerges in nearly all patients at this stage, progressively worsening as the disease advances, becoming a key clinical feature that accelerates disease progression. Furthermore, patients may present with proteinuria, hematuria, renal pain, kidney stones, urinary tract stones, urinary tract infections, intracranial aneurysms, heart valve abnormalities, and other complications. Notably, the incidence of intracranial aneurysms in ADPKD patients is approximately 12%, significantly contributing to early mortality [93].

In the advanced stages of ADPKD, the kidneys filled with numerous cysts, causing significant enlargement and an irregular shape, ultimately leading to declining renal function and potential renal failure in patients. In veterinary clinical practice, animals with clinical symptoms may exhibit loss of appetite, lethargy, dehydration, weight loss, increased thirst and urination, and reduced urine output or even anuria (Table 4, Figure 4 and Figure 5). Palpation of the kidneys may reveal enlargement and an irregular texture. However, these symptoms are nonspecific to ADPKD and can be observed in various other conditions. Some individuals, including dogs and cats, may remain subclinical without obvious symptoms. Blood indicators and biochemical parameters—such as urea, creatinine, and phosphorus—may remain within normal limits, especially at a young age.

Consequently, the diagnosis of ADPKD cannot rely solely on clinical manifestations or general physiological indicators like blood tests and urea levels. Additionally, it is imperative to consider other forms of cystic nephropathy that exhibit symptoms similar to those of ADPKD, including Autosomal Recessive Polycystic Kidney Disease (ARPKD), Nephonophthisis, and Autosomal Dominant Hereditary Renal Tubulointerstitial Nephropathy (ADTKD). ARPKD presents analogous symptoms to ADPKD such as renal failure, liver disease, and portal hypertension, albeit typically affecting children and young animals. Nephonophthisis and ADTKD have not been documented in veterinary clinics. In the human clinical context, Nephonophthisis is a hereditary renal condition characterized by tubulointerstitial damage, often manifesting as chronic nephropathy progressing to end-stage renal disease, with clinical features including progressive renal function deterioration or mild proteinuria. ADTKD is typified by renal interstitial fibrosis, presenting clinical signs such as progressive renal dysfunction, polyuria, polydipsia, anemia, and metabolic acidosis. Given the striking similarity in symptoms among these nephropathies, the diagnosis of polycystic kidney disease should not rely solely on clinical manifestations and general physiological indicators like blood and urea levels. Rather, monitoring and evaluating body state and renal function through these symptoms and indicators is crucial [94].

In human clinical practice, imaging studies are indispensable for diagnosing ADPKD, with kidney size assessment serving as the most reliable biomarker for predicting disease progression. Diagnostic criteria for individuals with a family history of ADPKD include the detection of at least three cysts in unilateral or bilateral kidneys in the 15–39 age group, at least two cysts in each kidney in the 40–59 age group, and at least four cysts in each kidney in the 60 and older age group, confirming an ADPKD diagnosis [95]. Another CT scan offering higher accuracy than ultrasound can be utilized. While ultrasound can detect cysts ranging from 0.5 to 1 cm, CT can identify cysts larger than 0.3 cm. In cases of unclear ultrasound findings or when molecular phenotyping is not feasible, magnetic resonance imaging (MRI) serves as an alternative to rule out other conditions. For individuals lacking a family history of ADPKD, a multifaceted diagnostic approach is necessary. During ultrasound examination, evaluation should extend beyond the kidneys to include organs like the liver, pancreas, and spleen. Provisional diagnosis of ADPKD can be made if more than 10 kidney cysts are detected alongside cysts in the liver, pancreas, or other organs. When the cyst count approaches the threshold and there is no significant increase in kidney size, ADPKD diagnosis can be confirmed through regular imaging to monitor cyst growth or via further genetic testing.

In veterinary clinical practice, ultrasound imaging serves as a practical tool for assessing the progression of ADPKD in dogs and cats. An ultrasound examination reveals numerous hypoechoic to anechoic cysts, characterized by thin or absent walls, circular or oval shapes, and clear demarcation from the surrounding renal parenchyma. Due to the lack of overt clinical signs in the early stages, early intervention is rare in dogs and cats. However, in advanced cases, ultrasound evaluation of total kidney volume and cyst volume is crucial for staging the severity of the disease. Ultrasound has proven to be an efficient method for detecting ADPKD of pets, with a sensitivity of 91% and a specificity of 100% at 36 weeks of age [96], cysts in the kidneys of cats can be detected as early as 7 weeks of age.

In addition to imaging examinations, genetic testing serves as a diagnostic tool for ADPKD. It is the preferred and reliable method for confirming the presence of mutations in genes associated with the disease and for early diagnosis. Genetic testing complements findings from CT scans, B-mode ultrasound, and MRI by providing genetic evidence. Through DNA analysis, mutations in genes linked to ADPKD, such as *PKD1* and *PKD2*, can be identified. This testing can be promptly conducted at any age or stage of disease. Early detection is preferred as it can effectively prevent the familial transmission of ADPKD and designing improved breeding scheme for cats or dogs carrying mutant genes. However, genetic testing is not yet widely performed, posing challenges for prevention and early intervention in ADPKD. In addition, cats and dogs typically do not undergo genetic testing before breeding, contributing to the rising number of ADPKD cases in these animals.

ADPKD is a chronic renal condition that typically manifests overt symptoms only in its later stages, posing significant challenges for diagnosis. By the time ADPKD is detected, it often has advanced to intermediate or advanced stages, precluding the opportunity for early intervention and treatment. In human or veterinary clinics, there is a widespread lack of awareness regarding the importance of early screening and monitoring for ADPKD. Therefore, establishing an effective screening protocol is crucial for the prompt diagnosis, treatment, and management of this condition.

## 6. Advancements and Challenges in ADPKD Treatment Strategies

ADPKD is a chronic renal condition frequently culminating in renal failure. Currently, there is no definitive cure for the disease, with treatment primarily centered on symptom management and supportive care. Although this approach can alleviate symptoms, it does not address the underlying condition. Drugs play a pivotal role in ADPKD treatment, with Tolvaptan emerging as a prominent therapeutic option. Tolvaptan, a selective vasopressin V2 receptor antagonist, has demonstrated efficacy in randomized clinical trials by attenuating the decline in renal function among ADPKD patients at high risk for rapid progression. To date, Tolvaptan remains the sole medication approved for ADPKD treatment [97]. Tolvaptan functions as a selective antagonist of the V2 receptor, regulating water reabsorption by modulating aquaporin-2 (AQP2) channels, concurrently, the inhibition of the cAMP pathway led to a decrease in the proliferation of renal tubular epithelial cells. When antidiuretic hormone (ADH)/vasopressin binds to the V2 receptor, it activates the cAMP signaling pathway, prompting the translocation of AQP2-expressing cells to the apical plasma membrane, thus enhancing water reabsorption [98]. The blockade of V2 receptors effectively inhibits the signaling cascade of the cAMP pathway and the actions of ADH. Consequently, the expression and activity of AQP2 are suppressed, leading to a reduction in water reabsorption by the renal tubules. This decrease in water reabsorption is accompanied by an increase in the plasma concentration of Na^+^. These combined effects enhance the kidney’s capacity to manage water balance, promoting the excretion of excess water through urine and concurrently diminishing the production of fluid. However, Tolvaptan is associated with various adverse effects, including polyuria, thirst, dry mouth, nausea, vomiting, headache, and fatigue. It may not be appropriate for individuals with renal impairment or those intolerant to the medication. In addition, Tolvaptan can interact with other drugs, particularly those affecting liver metabolic enzymes [99]. Given these limitations, there is a necessity to explore strategies that either mitigate adverse reactions or enhance the efficacy of ADPKD treatment. Due to the limitations inherent in the treatment of Tolvaptan, it is imperative to explore alternative approaches to mitigate adverse reactions in patients and to develop more effective treatments for ADPKD. Extensive research has identified and validated that agents such as sirolimus [100] and rapamycin [101] can exert beneficial effects on the murine model of ADPKD. These drugs demonstrate the ability to slow cyst growth by inhibiting the mTOR signaling pathway. However, they have yet to successfully navigate clinical trials, and their efficacy for human ADPKD patients remains uncertain. Consequently, there is a compelling need to shift focus to inter-species research. Given the anatomical and functional similarities between porcine and human kidneys, the porcine model may offer a more suitable platform for investigating ADPKD pathogenesis and therapeutic strategies.

Other than medications, surgical intervention represents a conventional approach to managing ADPKD, involving several procedures like cyst decaplication and decompression; capsulotomy, puncture and sclerosis; kidney transplantation; and nephrectomy [102]. For a solitary large cyst, a surgeon can perform a small incision into the cyst, drain the fluid, and then close the wound. This method can alleviate cystic pressure and potentially enhance renal function. While surgical interventions can effectively alleviate the symptoms associated with polycystic kidney disease, they are not without significant risks. The procedure itself carries a high operational risk, with the potential for damage to normal tissues during surgery. Additionally, postoperative complications such as urinary tract obstructions are not uncommon. It is important to note that these treatments address the symptoms rather than the underlying cause of the disease, and there is a heightened risk of cyst recurrence. In cases of severe renal impairment caused by numerous cysts, the consideration of nephrectomy or kidney transplantation may arise. A recent study evaluated the intraoperative assistance of three-dimensional virtual models (3DVMs) in highly complex minimally invasive partial nephrectomy for renal tumors [103], highlighting their potential value in improving surgical accuracy and postoperative recovery for patients. This may also offer insights for the resection of renal cysts. Through kidney transplantation, the patient’s symptoms can indeed be alleviated. However, the potential complications and risks of rejection associated with the transplant cannot be ignored. Moreover, kidney transplantation only addresses the issue of kidney cysts; if the patient has cysts in other areas, the transplant will not resolve these issues. Nonetheless, these procedures are intricate, donor kidneys are scarce, and the associated costs are prohibitive, rendering widespread implementation challenging.

Renal replacement therapy is imperative for patients with ESRD. Given the enlarged abdomen and diminished effective peritoneal area in ADPKD patients, hemodialysis typically prevails over peritoneal dialysis as the preferred choice. However, a retrospective cohort study has indicated no substantial disparity in 5-year survival rates or peritonitis risk between patients undergoing peritoneal dialysis and those receiving hemodialysis [97]. Consequently, peritoneal dialysis stands as a viable treatment alternative for ADPKD patients with ESRD.

With the increasing knowledge of ADPKD, novel treatment strategies continue to emerge. Promising drugs like octreotide-LAR, somatostatin [104] and hydrochlorothiazide [105] are currently in development, offering potential additional options for ADPKD management. In particular, the hydrochlorothiazide has been shown to mitigate the polyureic effect associated with Tolvaptan in patients. Moreover, it significantly reduces multiple markers indicative of kidney damage and dysregulation in glucose metabolism within a murine model [105]. Gene therapy constitutes a burgeoning frontier in research, exemplified by innovative treatments like Casgevy. Casgevy, an ex vivo gene editing therapy, utilizes CRISPR/Cas9 technology to modify hematopoietic stem cells. This groundbreaking treatment is a collaborative effort between Vertex Pharmaceuticals, an American biotechnology firm, and CRISPR Therapeutics, a Swiss pharmaceutical company. The approval of Casgevy by the FDA marks a significant milestone in gene therapy [106,107,108,109,110]. Concurrently, stem cell therapy is emerging as a rapidly evolving field, particularly in renal disease research. Stem cell therapy approaches include direct stem cell differentiation into renal parenchymal cells, enhancement of renal secretion, mitigation of inflammatory responses, and restoration of renal blood circulation, all contributing to renal injury repair and kidney function improvement. As stem cell research technology progresses, further advancements are anticipated in understanding stem cell differentiation into kidney cells, thereby broadening the therapeutic scope of stem cell transplantation in treating kidney diseases [111,112,113,114,115].

## 7. Advancements in Animal Models for ADPKD Research

Animal disease models play a crucial role in the fields of medicine and life sciences, aiding in the comprehensive understanding of disease etiologies and pathophysiological mechanisms. They serve as a cornerstone for the formulation of preventive and therapeutic strategies. Additionally, these models are indispensable for evaluating the effectiveness and safety of novel pharmacological interventions and treatment approaches. The controlled laboratory setting enables meticulous assessment of drug safety, efficacy, and potential adverse reactions, thus yielding invaluable insights for therapeutic intervention. Furthermore, through the integration of genetics and disease prediction methodologies, animal models contribute significantly to the advancement of innovative gene therapies. Therefore, animal disease models represent an essential tool for unraveling the complexities of various diseases, serving as a crucial platform for medical progress.

At present, there is a dearth of established animal models for ADPKD in canines and felines, although relevant models exist in murine, rodent, and miniature porcine species. Currently, two primary categories of animal models for ADPKD prevail. The first comprises spontaneous genetic models exhibiting characteristic ADPKD phenotypes, while the second involves models generated through homologous gene mutations mirroring those found in humans. Published studies have elucidated several genes implicated in spontaneous genetic models, especially, Cys1, Nphp3, Anks6, and Pkhd1, among others, which are associated with renal cystogenesis in cpk mice, pcy mice, Han: SPRD cy rats, and PCK rats [116,117,118,119]. It has been observed that these gene products also correspond to the PC-1 and PC-2 proteins [116,117,118,119].

To advance our understanding of ADPKD pathogenesis, a multitude of genetically modified animal models have been developed. Initially, ADPKD mouse models predominantly employed traditional homologous recombination techniques to knock out the *PKD1* or *PKD2* genes. Presently, the Cre-loxP system enables precise gene modifications in specific organs by flanking the target region with loxP sites. The *Ksp-Cre; PKD1^flox/flox^* model involves breeding mice carrying a single loxP gene locus on each side of the target area with mice that express Ksp-Cre to create a kidney-specific knockout model of *PKD1*. Using this model, people have carried out a variety of studies, finding that cysts originate from cells with active Cre recombinase, accompanied by activated MAPK/ERK signaling. Nonetheless, inhibition of ERK1/2 activation fails to impede cyst growth [120]. The *Ksp Cre; Pkd1^flox/flox^* mouse model also served as a platform to evaluate mitochondrial abnormalities associated with ADPKD. The observed accumulation of mitochondrial abnormalities in this ADPKD model lends support to the role of disrupted Ca^2+^ influx and increased cAMP levels in ADPKD progression [121,122]. In addition, the human *PKD1* mutant p.Arg3277Cys (RC) represents a sub-allelic variant, and introducing this mutation into mice (as *Pkd1^RC/RC^*) can attenuate the progression of ADPKD in these animals. In contrast to typical homozygous ADPKD models, the *Pkd1^RC/RC^* model can induce slowly progressed ADPKD and has been extensively utilized in preclinical investigations [123]. A study utilizing the *Pkd1^RC/RC^* mouse model to explore Tolvaptan treatment demonstrated a reduction in kidney weight-to-body weight (KW/BW) ratio in Tolvaptan-treated mice, along with decreased volume and number of cysts, accompanied by significant reductions in blood urea nitrogen and cAMP levels [124]. Another model, the doxycycline-induced *Pax8^rtTA^; TetO-Cre; Pkd1^flox/flox^* model, enabling spatial-temporal control of gene activity, presents a progressively slower and more human-like ADPKD phenotype. In this model, rtTA is exclusively expressed in kidney cells under the control of the Pax8 promoter. Upon activation, rtTA binds to the TetO sequence, thereby initiating the transcription of the Cre gene. The Cre recombinase, once produced, recognizes and recombines the loxP sites flanking the *Pkd1* gene, leading to its specific knockout in kidney cells. The expression of TetO-Cre is regulated by tetracycline administration, by manipulating the timing and duration of tetracycline treatment, researchers can precisely control when the *Pkd1* gene is knocked out, allowing for the study of its effects on kidney function at defined stages [125]. Researchers recently utilized the *Pax8^rtTA^; TetO-Cre; Pkd1^flox/flox^* model to explore the therapeutic potential of 11 beta-dichloro in ADPKD. Their study demonstrated that 11 beta-dichloro significantly improved renal structure and function in mice of this model, without any indication of significant toxicity [126].

In PKD, mutations classified as non-protein truncating and protein truncating significantly impact the average age of onset for renal failure [127]. This variability is attributed to individual differences, genetic backgrounds, lifestyle factors, and disease management strategies. Non-protein truncating mutations, such as missense mutations, can cause partial functional impairment of PC-1 or PC-2, resulting in partial protein activity. Consequently, these mutations are associated with a slower disease progression, with renal failure typically occurring later in middle or later adulthood. Conversely, protein truncating mutations, including nonsense mutations, alterations in splicing sites, or large deletions, lead to the premature termination of polycystin synthesis, resulting in non-functional protein fragments or complete absence of the protein. These truncating mutations are associated with more severe disease manifestations and accelerated progression, leading to an earlier onset of renal failure, potentially in early adulthood or middle age. Specifically, patients with *PKD1* gene mutations, particularly those that are truncated, may experience ESRD between the ages of 30 and 50. In contrast, *PKD2* gene mutations typically result in a slower disease progression, with ESRD typically occurring after the age of 50 or at a later stage.

Miniature pigs and monkeys are widely accepted large animal models in human disease modeling. Our group have established a *PKD2* transgenic miniature pig model [127]. Despite the initial high expression of the *PKD2* gene observed through PCR and Western blot analyses, early stages of ADPKD progression did not manifest evident pathological alterations, and there were no significant differences in urea nitrogen and creatinine levels between wild-type and transgenic pigs. Therefore, continued monitoring is essential to detect any potential pathological changes in the kidneys of transgenic pigs. Subsequently, our group [128] utilized the first-generation genome editing technology, zinc-finger nucleases (ZFNs), to establish a miniature pig model with a single-allele knockout of *PKD1*. By approximately 6 months of age, cyst formation commenced in the pig kidneys, exhibiting a more rapid growth compared to the previously established polycystic kidney model of transgenic miniature pigs with *PKD2*, as well as faster progression than ADPKD mice with a single-allele knockout of *PKD1* (*PKD1^+/−^*). Masahito Watanabe and colleagues [129] recently developed a *PKD1^insG/+^* pig model using the CRISPR-Cas9 gene editing technique. Investigations have revealed that newborn piglets in the *PKD1^insG/+^* model demonstrate renal cysts and interstitial fibrosis, accompanied by an early asymptomatic phase, mirroring the characteristics observed in ADPKD patients with *PKD1* heterozygous mutations. Tomoyuki Tsukiyama et al. [130] pioneered the creation of an ADPKD model with a *PKD1* mutation in crab-eating macaques using CRISPR/Cas9. Heterozygous crab-eating macaques manifested a small number of cysts either at birth or around 2 months of age, predominantly located in the distal tubules, resembling the scenario observed in human heterozygous pediatric patients. Utilizing monkey models for studying the cyst formation process proves to be more practical than relying solely on mice, which necessitate conditional knockout strategies to mimic human phenotypes. Nevertheless, the significant time and financial investments associated with using monkeys as model organisms must be duly considered.

To date, the mouse model has been extensively employed across various disciplines, encompassing immunology, infectious diseases, cancer biology, and drug research, significantly augmenting our understanding of ADPKD. However, many mouse disease models inadequately represent the human internal environment, and some exhibit excessively rapid disease progression, diverging starkly from the pace observed in humans. This discrepancy is particularly pronounced in ADPKD, where mouse models often demonstrate a rapid disease onset and progression, leading to the development of drugs with limited efficacy in humans. Consequently, there is an urgent need for disease models that more accurately mirrors human physiological structure and disease progression. Among the available options, the pig disease model emerges as an alternative. Pigs exhibit a high degree of anatomical and physiological similarity to humans, including weight, organ formation, and disease occurrence, without any artificial manipulation. Additionally, mice possess a unilobular kidney and are substantially smaller than human kidneys, comprising only about one-fifth of their size, with fewer nephrons. In contrast, both pigs and humans possess multiple renal papillae with comparable morphology, size, and anatomical structure. A comparative assessment of kidneys across various species underscores that pigs and humans exhibit greater morphological, size, and physiological functional resemblances than other animal models (Table 5) [131,132,133,134,135,136,137].

Moreover, pigs exhibit a remarkable degree of genomic and chromosomal resemblance to humans. Their genome size, structure, number, and chromosomal arrangement closely parallel those of humans, accompanied by a plethora of shared gene sequences. With the advent of gene editing technologies, traditional homologous recombination methods and cutting-edge nuclease technologies—such as ZFNs, Transcription Activator-Like Effector Nucleases (TALENs), and the CRISPR-Cas9 system [109,110]—alike have significantly advanced. Concurrently, there has been rapid progress in generating transgenic models, gene knockout/knock-in models, and pigs bearing precise gene modifications [111]. Cloning techniques have become more mature compared to before, while the establishment of cell lines has become relatively facile, bolstering the development of gene-editing models in miniature pigs. As pig genome sequencing continues to refine, researchers will gain access to more potent genetic and proteomic tools tailored for pig-specific research endeavors. Furthermore, ethical concerns associated with pigs are comparatively fewer than those associated with mice, dogs, cats, and monkeys. Thus, utilizing pig disease models offers substantial advantages for investigating pathological mechanisms and therapeutic strategies applicable to both animal and human contexts.

## 8. Conclusions

Research on polycystic kidney disease has been ongoing for decades. Currently, significant advancements have been achieved in understanding the pathological changes, molecular mechanisms, and diagnosis of human ADPKD. The progress in molecular genetics and imaging techniques has remarkably enhanced the accuracy of diagnosis. However, it is often overlooked that polycystic kidney disease not only has a high incidence and heredity in humans, but also in other animals, such as dogs, cats, pigs, cattle, and sheep. The genetic patterns and molecular mechanisms of polycystic kidney disease among different species are highly similar. For instance, ADPKD in humans, dogs, and cats is all caused by a single gene mutation, and polycystic kidneys in domestic animals such as pigs are often confined to the same populations as humans. Human ADPKD shares many similarities in pathological and clinical features with animals such as dogs, cats, and pigs, including the occurrence of cysts and impaired renal function in the kidneys. However, due to species and individual differences, there is still a great deal of complexity and diversity. Therefore, when conducting research on polycystic kidney disease, it is important to note that the incidence, cycle, and degree of ADPKD vary among different species, with small pigs being the closest to humans. When studying genes related to polycystic kidney disease, these different manifestations can be combined to identify gene mutations and further explore the diverse pathogenesis of polycystic kidney disease. At present, there is no method or drug that can cure polycystic kidney disease. Only Tolvaptan is approved for the treatment of human ADPKD, and there are no suitable drugs available for use in veterinary clinical practice or animal husbandry. It is time for us to explore alternative research methods. Presently, there are numerous mouse models available, yet it is evident that pig models offer closer parallels to humans in terms of function, structure, and response. They can better replicate human disease characteristics. The gene-edited pig model, established through genetic editing in pigs, has emerged as one of the most favored large animal models. Perhaps shifting our research focus from mice to small pigs could yield significant breakthroughs in ADPKD research.

## Figures and Tables

**Table 1:** Pathological Characteristics of ADPKD in Humans, Dogs, Cats, and Livestock.

Species	Human	Dog	Cat	Livestock
Cyst Formation	Kidneys, liver	Kidneys, liver	Kidneys, liver	Kidneys, liver
Additional Organ Involvement	Liver, pancreas, spleen, thyroid	Liver, pancreas, uterus, abdominalcavity	Liver, pancreas, uterus, abdominal cavity	Ascites, bile ducthyperplasia
Histological Features	Interstitial inflammation, fibrosis, EMT, ECM accumulatione	Proliferation of renal Tubular epithelium, heartdisease	Increased arterial pressure, proliferation ofcyst lining epithelial cells	Atrophy, degeneration, fibrous connective tissue, cyst formation
Clinical Progression	Chronic renal failure, hypertension, cardiovascular complications	Gradual deterioration, end-stage renal disease	Early renal failure, variecsymptoms, end-stage renal disease	Organ enlargement, reduced production efficiency

**Table 2:** Molecular Characteristics of ADPKD in Humans, Dogs, Cats, and Livestock.

Species	Human	Dog	Cat	Livestock
Genetic Factors	Shared gene mutations, familial inheritance	Familial inheritance, breed-specific mutations	Familial inheritance, breed-specific mutations	Familial inheritance, breed-specific mutations
Research Approaches	Genetic sequencing, geneexpresslon analysis	Comparative genomics, histological studies	Genetic testing	Genetic mapping, histopathological analysis
Commonalities Identified	Pathways involving fibrosis, ECM accumulation	Renal tubular epithelial cell proliferation	Arterial pressure increase, cystlining epithelial cell proliferation	Renal and liver cyst formation, fibrous connective tissue

**Table 3:** Summary of Mutations Associated with ADPKD Across Species.

Species	Human	Dog	Cat	Livestock
Gene	*PKD1* *PKD2*	*PKD1*	*PKD1*	Unknown
Mutation	Various deletions, insertions, substitutions in *PKD1* gene	G>A mutation on chromosome 29	C.10063C>A mutation in exon 32	Unknown mutations
Phenotypic Effect	Precipitation of symptom manifestation, contribution to ADPKD development	Associated with bull terrier polycystic kidney disease (BTPKD)	Premature termination codon, loss of 25% of PC-1 C-terminus	Resemble human ADPKD symptoms and pathology

**Table 4:** Comparison of Diagnostic Approaches for ADPKD in Human and Veterinary Clinical Practice.

Diagnostic Method	Human Clinical Practice	Veterinary Clinical Practice
Clinical Symptoms	Headaches, insomnia, blurred vision, hypertension, proteinuria, hematuria, renalpain, kidney stones, urinary tract infections, intracranial aneurysms, heart valve abnormalities	Loss of appetite, lethargy, dehydration, weight loss, increased thirst andurination, reduced urine output, anuria, palpable kidney enlargement and irregular texture
Imaging studies	Ultrasound, CT scanning, MRI	Ultrasound
Biomarkers	Blood indicators: urea, creatinine, phosphorus	Blood indicators: urea, creatinine, phosphorus
Genetic Testing	Identifies mutations in *PKD1* and *PKD2* genes	Identifies mutations in *PKD1* and *PKD2* genes
Diagnostic Criteria	Based on cyst counts on ultrasound, family history	Based on cyst counts on ultrasound, family history

**Table 5:** Renal Structure and Physiological Indexes of Different Species.

Structure and Index	Human	Pig	Dog	Mouse
Kidney weight	120–150 g	150 g	39 g	0.176 g
Renal volume	120–490 cm^3^	38–118 cm^3^	20–34 cm^3^	0.34 cm^3^
Renal papilla	Multipapilla	Multipapilla	Monopapilla	Monopapilla
Bilateral nephron	2–3 million	2.2 million	0.8 million	30,000
Glomerular filtration rate	80–120 mL/min	100 mL/min	61.3 mL/min	0.28 mL/min
Filtration fraction	0.20	0.24	0.32	0.23
Relative medullary thickness	3.0	1	4.3	5.6
Urine pH	4.5–8	6.25–7.55	6–7	7.3–8.5
24-h urine volume	1–2 L	2–5 L	0.25–1 L	0.001–0.003 L
